# Pupil light reflex dynamics in Parkinson’s disease

**DOI:** 10.3389/fnint.2023.1249554

**Published:** 2023-08-31

**Authors:** Panagiota Tsitsi, Mattias Nilsson, Josefine Waldthaler, Gustaf Öqvist Seimyr, Olof Larsson, Per Svenningsson, Ioanna Markaki

**Affiliations:** ^1^Department of Clinical Neuroscience, Neuro, Karolinska Institutet, Stockholm, Sweden; ^2^Center for Neurology, Academic Specialist Center, Stockholm, Sweden; ^3^Department of Clinical Neuroscience, Eye and Vision, Karolinska Institutet, Stockholm, Sweden; ^4^Division of Aging Research Center, Karolinska Institutet, Stockholm, Sweden; ^5^Department of Neurology, Karolinska University Hospital, Stockholm, Sweden

**Keywords:** pupil reflex, Parkinson’s disease, eye movements, eye tracking, dysautonomia

## Abstract

**Introduction:**

Visual disturbance is common symptom in Parkinson’s disease (PD), and defective pupil light reflex (PLR) is an anticipated contributing factor that may be associated to the presence of autonomic dysfunction, which is a common non-motor feature of PD. Studies investigating the intercorrelation between PLR and dysautonomia in PD are limited.

**Methods:**

The aim of this study was to investigate differences of PLR parameters, measured by eye-tracker, between patients with PD, with and without signs of dysautonomia, and healthy controls (HC). In total, 43 HC and 50 patients with PD were recruited and PLR parameters were measured with Tobii Pro Spectrum, during a long (1,000 ms) and a short (100 ms) light stimulus. Presence of orthostatic hypotension (OH) was used as proxy marker of dysautonomia. Linear mixed-effects model and non-parametric comparative statistics were applied to investigate differences among groups.

**Results:**

Peak constriction velocity was slower in PD compared with HC, after adjustment for age and sex in the mixed model, and the difference was greater in the subgroup of PD with OH (unadjusted). Dilation amplitude and velocity were also gradually slower in HC vs. PD without OH vs. PD with OH (unadjusted for confounders). In the mixed model, age was significant predictor of dilation response.

**Discussion:**

Our results support previous observations on defective PLR in PD, evaluated with eye-tracker, and show a possible association with autonomic dysfunction. Further studies with more patients and rigorous evaluation of autonomic dysfunction are needed to validate these findings.

## 1. Introduction

Autonomic dysfunction (dysautonomia) is a common feature in Parkinson’s disease (PD). Orthostatic hypotension (OH) together with decreased heart rate variability, thermoregulation malfunction, nocturia and urinary urgency, impotence, constipation and delayed gastric emptying, as well as dysphagia, are common clinical manifestations in PD that are related to dysautonomia (Metzger and Emborg, 2019). On the other hand, visual complaints like diplopia, defective visual acuity, abnormal eye movements, decreased blink frequency and visual hallucinations are also common, along with abnormal pupil light reflex (PLR). However, although most of the aforementioned disturbances have been related to dopamine loss in the retina and the central nervous system, PLR defects are mainly attributed to dysautonomia (Armstrong, 2015). Both the parasympathetic and sympathetic system play an important role in the regulation of the PLR, and imbalance between the two results in abnormal pupil reactivity that can occur early in the course of the disease (Armstrong, 2015).

Pupil size, dynamics, and responsivity provide insights on the integrity of the neural system. The role of the autonomic nervous system (ANS) in the regulation of PLR is well known. The parasympathetic pathway is responsible for pupil constriction (miosis) while the sympathetic pathway controls pupil dilation (mydriasis). The PLR is primarily driven by changes in luminosity, but it is also affected by other parameters such as cognitive factors, stressful events, medication, and various diseases (Ciuffreda et al., 2017). Most of the nerve fibers originating from the retinal ganglion synapse in the lateral geniculate nuclei are part of the visual pathway. A small number of fibers, however, travels to the hypothalamus and the olivary pretectal nucleus (OPN). OPN, in turn, provides efference for the PLR both with regards to the parasympathetic and the sympathetic systems, respectively (Ciuffreda et al., 2017; Yoo and Mihaila, 2020). Meanwhile, the Edinger–Westphal nucleus plays a dual role taking part in both the parasympathetic and sympathetic functions, since it comprises two separate cell populations. While the first population is part of the parasympathetic pathway, the second population comprises peptidergic centrally projecting neurons involved in stress adaptation and sympathetic functions (Kozicz et al., 2011; Heiland Hogan et al., 2020).

Changes in pupil constriction and dilation in PD have previously been described (Yamashita et al., 2010) and they could be attributed to altered pathology of various neural formations, such as the Edinger–Westphal nucleus or the superior cervical ganglion (Hunter, 1985; Del Tredici et al., 2010). Moreover, *in vitro* study findings on donor irises have shown that PD patients’ iris dilator and sphincter muscles develop adaptive changes in the form of hypersensitivity and therefore increase their maximum contraction ability (Patil and Mauger, 1992).

The aim of the study was to investigate differences in transient PLR in persons with PD compared to healthy controls (HC) and relate to OH as a hallmark of dysautonomia.

## 2. Materials and methods

### 2.1. Participants and clinical evaluation

Fifty PD patients of an early stage (Hoehn and Yahr ≤3) and 43 healthy volunteers were recruited at the Center for Neurology, Academic Specialist Centrum in Stockholm, Sweden, as described previously (Tsitsi et al., 2021, 2023). The study was approved by the Stockholm Ethical Committee (DNR: 2018/437-31/2) and participants provided written and oral informed consent. Individuals suffering from pathological eye conditions were excluded and vision was normal or corrected to normal. Disease duration and levodopa equivalent daily dosage (LEDD) were determined in the PD group, and all participants were evaluated with the Unified Parkinson’s Disease Rating Scale (UPDRS). For the purpose of the study, we further divided the PD group into two separate groups according to the presence or absence of orthostatic symptoms, based on the patients’ records. In those cases where formal orthostatic testing was not available from diagnosis until one year after eye-tracking measurements, classification was based on patients’ subjective reporting on the presence of OH symptoms (*n* = 6). Due to bad quality of recordings, 16 participants were excluded from final statistical analysis. Additionally, we excluded six recordings of patients that were diagnosed with conditions (diabetes mellitus), or treated with concomitant medication (anticholinergics, cholinesterase inhibitors, and glaucoma treatment), that could interfere with the pupil size evaluation. Totally, 71 participants (35 HC and 36 PD) were included in the final analysis.

### 2.2. Eye tracker and paradigms

Eye position and pupil size were measured with Tobii Pro Spectrum (examples of raw data shown in Supplementary Data Sheet 1), a screen-based eye tracker with an acquisition speed of 1,200 Hz. Instructions were provided by the examiner and a calibration of the system was performed in the beginning and as often as needed to ensure optimum quality of recordings. The duration of the task was 2.8 min, excluding participant instructions and eye tracker calibration. To reduce potential effect of the circadian rhythm, all measurements were performed between 11:00 and 15:00. Visual stimuli were displayed on a 24” screen located approximately 65 cm in front of the participant who was sitting comfortably in a still chair throughout the test in a dimly lit room. The participant was instructed to keep his or her eyes focused on a dot in the center of the screen. Following a brief appearance of a black fixation cross on white background, black background with a central gray fixation point were displayed for 15 s (pre-stimulus period). Then the screen was illuminated (luminance of 250 cd/m^2^) by a brief and sudden bright white “flash” for a duration of 100 ms (short stimulus), which was followed by a 15 s post-stimulus response period (gray fixation point centered on black background). At the end of this period, the screen was illuminated again by a bright white “flash” for a duration of 1,000 ms (long stimulus) followed by a 15 s post-stimulus response period. The short and long stimuli along with the subsequent 15 s post-stimulus response period were alternated five times, giving five repetitions per condition in total.

Data were filtered through the Tobii Identification by Velocity Threshold (I-VT) algorithm available in the Tobii Pro Lab software to accurately identify fixations and saccades in the raw gaze data. More details on the process can be found in our previous publication (Tsitsi et al., 2021).

We examined the pupillary response in two conditions, after a short and a long flash, that were expected to elicit different timing and magnitude of the pupillary response. All stimulus-response parameters were computed over a 7 s window following stimulus onset, based on the mean pupil diameter of the left and right eye. The parameters were first computed for each participant and repetition, and then “averaged” across the five repetitions in each condition by taking the median of the repetitions. Repetitions in which the percentage of noise samples in the constriction phase exceeded 40% of the gaze samples were excluded. Thus, all computed values are based on at least 60% (i.e., a majority) of the original and non-interpolated data. Pupil size during the pre-stimulus and stimulus-response period, and measures of pupil dynamics in the constriction and the dilation phase were computed (Supplementary Tables 1, 2). Comparisons and correlations between left and right pupil measurements, blink frequency and noise levels/data quality in the original non-interpolated data were also determined.

### 2.3. Statistical analysis

Demographic and clinical characteristics were compared using the χ^2^ test to assess differences in categorical variables, and the non-parametric Mann–Whitney U test and nptrend test (Stepniewska and Altman, 1992) were used to assess differences of continuous variables; Stata 16.0.

Additionally, we used R programming language (4.2.1) with the lmer4 and lmerTest packages (Bates et al., 2015). For the comparison between HC and PD groups, all pupil measures were first scaled to a mean of zero and standard deviation of one and then analyzed using generalized mixed-effects linear models with group (HC/PD), age, and sex as independent variables and subject as a random effect. *T* tests with Satterthwaite’s approximation to the degrees of freedoms were used (Satterthwaite, 1946; Welch, 1947). Statistical significance was asserted at α = 0.05.

## 3. Results

### 3.1. Participants’ characteristics

In total, 36 PD patients and 35 HCs were included in the analysis. Sex distribution differed with more men included in the PD than the HC group (*p* = 0.004), whereas age did not differ between groups (Table 1). Median disease duration in the PD group was 2 years, corresponding to early-stage disease, as indicated also by the rather low median LEDD (545 mg) and UPDRS part 3 score (18.5 points) (Table 1). Patients with PD and OH (*n* = 6) comprised 5 (83%) males, and they were older than HC and PD without OH (median 71, 62, and 64 years respectively; *p* = 0.1). Also, patients with PD and OH tended to have higher LEDD and UPDRS motor and total scores than PD patients without OH (Table 1).

**TABLE 1:** Clinical and demographic characteristics of the study population.

	HC	PD	*p*	PD without OH (*n* = 30)	PD with OH (*n* = 6)	*p*
Sex, M/F	11/24	24/12	**0.004**	19/11	5/1	0.3
Age, years	62 (16)	65 (11.5)	0.4	64 (11)	71 (6)	0.1*
Years since diagnosis		2 (3)		2.5 (3)	2 (4)	0.9
LEDD		545 (475)		521 (440)	812 (525)	0.2
UPDRS part 3		18.5 (15.75)		18 (15)	26.5 (16)	0.5
UPDRS total		32.5 (24.5)		31 (30)	40.5 (24)	0.6

Values are reported as medians and interquartile ranges (IQR), apart from the gender ratio that is reported in absolute values. Statistical significant (*p* < 0.05) results are highlighted in bold. M, male; F, female; HC, healthy controls; PD, Parkinson’s disease; LEDD, levodopa equivalent daily dosage; OH, orthostatic hypotension; UPDRS, Unified Parkinson’s Disease Rating Scale.

*p-Value for the comparison between HC, PD with and PD without orthostatic hypotension.

### 3.2. Effect of sex, age, and disease state on stimulus-response parameters

A significant effect of sex on blink frequency was identified in both the short (β = −0.596, SE = 0.194, *t* = −3.065, *p* = 0.003) and long (β = −0.500, SE = 0.199, *t* = −3.273, *p* = 0.002) light condition (Supplementary Table 3), confirming existing evidence that females blink more frequently compared to males (Sforza et al., 2008). The linear mixed-effects model showed a significant effect of age on several dilation parameters both in the short and long light conditions (Supplementary Table 3). Furthermore, peak constriction velocity was slower for the PD group compared to HC after adjustment for age and sex, both in the short- and long-flash conditions (β = 0.428, SE = 0.194, *t* = 2.208, *p* = 0.03 and β = 0.496, SE = 0.2094, *t* = 2.368, *p* = 0.02, respectively). Dilation amplitude and velocity in the short-flash condition tended to be smaller and slower, respectively, in the PD group, but the difference did not reach statistical significance in the mixed model (*p* = 0.08 and *p* = 0.06, respectively).

### 3.3. Effect of OH on stimulus-response parameters

In our *post hoc* analysis, we found differences between HC, PD without OH and PD with OH on peak constriction velocity, constriction slope, dilation amplitude, dilation velocity in the short-flash condition, as well as peak constriction velocity, constriction slope, dilation amplitude and dilation velocity and in the long-flash condition [nptrend (Stepniewska and Altman, 1992); Stata 16.0; Table 2]. In these comparisons, PD patients with OH presented with slower and less effective pupil dynamics in both light conditions.

**TABLE 2:** Pupil response metrics recorded during the short (100 ms flash stimulus) and long (1,000 ms flash stimulus) light conditions.

	HC (*n* = 35)	PD without OH (*n* = 30)	PD with OH (*n* = 6)	*p*	*p* for all PD vs. HC
**Short flash**					
Peak constriction velocity	−4.85 (1.05)	−4.23 (0.95)	−4.05 (0.91)	**0.02**	**0.02**
Constriction velocity	−1.8 (0.42)	−1.53 (0.47)	−1.59 (0.33)	0.09	0.06
Constriction slope	−0.002 (0.0004)	−0.0017 (0.0005)	−0.0018. (0.0004)	0.08	**0.04**
Dilation amplitude	1.5 (0.4)	1.2 (0.6)	1.1 (0.4)	**0.02**	**0.03**
Dilation velocity	0.24 (0.07)	0.2 (0.1)	0.19 (0.08)	**0.02**	**0.03**
**Long flash**					
Pupil diameter SD	0.52 (0.17)	0.42 (0.2)	0.42 (0.1)	0.06	0.05
Constriction latency	371 (376)	560 (286)	624 (350)	0.06	0.08
Peak constriction velocity	−4.8 (1.05)	−4.1 (1.25)	−3.9 (1.4)	**0.003**	**0.002**
Time to peak constriction velocity	388 (44)	401 (71)	412 (158)	0.05	0.07
Constriction slope	−0.0015 (0.0004)	−0.0012 (0.0005)	−0.0014 (0.0003)	0.06	**0.01**
Dilation amplitude	1.7 (0.6)	1.4 (0.7)	1.3 (0.4)	**0.02**	**0.02**
Percentage change of dilation amplitude	66.9 (17)	55.5 (25)	60.9 (15)	0.09	0.06
Dilation velocity	0.26 (0.08)	0.21 (0.09)	0.2 (0.05)	**0.04**	**0.04**
Dilation slope	0.00015 (0.00008)	0.00012 (0.00009)	0.00012 (0.00005)	0.07	0.06
Dilation intercept	3.1 (0.5)	2.9 (0.5)	2.7 (0.8)	0.09	0.1

Comparisons between HC, PD without OH and PD with OH. Values are reported as medians and interquartile ranges (IQR). Statistical significant (*p* < 0.05) results are highlighted in bold. HC, healthy control; PD, Parkinson’s disease; OH, orthostatic hypotension; SD, standard deviation. Pupil diameter SD is the standard deviation of the pupil size during the stimulus-response period computed in mm. Amplitudes are computed in mm. Velocities are computed in mm/s. Latency and time to peak constriction velocity are computed in ms. The constriction latency is estimated using a change-point detection model which identifies the first point in time at which the statistical properties (mean and variance) of the signal changes significantly.

No correlation was observed between these PLR parameters and age, LEDD, UPDRS motor or total score, as assessed with Spearman correlations in the PD group. LEDD score over 787 mg (75th percentile) was used to define groups with high vs. low LEDD, and no differences were observed in the above PLR parameters between groups.

### 3.4. Effect of stimulus type on pupil size dynamics – interaction with disease state

There is strong linear correlation between amplitude and peak constriction velocity in healthy persons (Bremner, 2012), which we could confirm in our data as well, both in the long-flash and short-flash condition paradigm (Table 3).

**TABLE 3:** Spearman rho between amplitude and peak constriction velocity in the short and long light condition in the four groups, namely HC, PD total, PD without and PD with orthostatic hypotension.

	Short	Long
HC	0.668 (*p* < 0.001)	0.838 (*p* < 0.001)
PD (total)	0.931 (*p* < 0.001)	0.969 (*p* < 0.001)
PD without OH	0.955 (*p* < 0.001)	0.971 (*p* < 0.001)
PD with OH	0.829 (*p* = 0.04)	0.943 (*p* = 0.005)

HC, healthy control; PD, Parkinson’s disease; OH, orthostatic hypotension.

Interestingly, we observed also a significant within-group difference with prolonged PLR latency in the long flash paradigm compared to the short light condition in all three groups (median 371 vs. 249.17 ms in the HC group, *p* < 0.001, 560.8 vs. 252.9 ms, *p* < 0.001, in the PD group without OH and 623.8 vs. 265 ms, *p* < 0.001 in the PD with OH group).

## 4. Discussion

Our study shows that PLR can be robustly evaluated with the more easily applicable eye-tracker, compared to pupillometer, and our results indicate that both miosis and mydriasis are affected in PD patients, compared to healthy participants. The defect in peak constriction velocity is more prominent in patients that suffer peripheral autonomic dysfunction, defined by the presence of OH. In a previous systematic review, peak constriction velocity was suggested as a sensitive parameter of parasympathetic dysfunction (Wang et al., 2016).

A study on unselected patients with generalized dysautonomia, not including PD patients, showed that only 2/3 of the cohort presented with a variety of pupil abnormalities, which indicates that dysautonomia *per se* is not related with PLR dysfunction in an absolute manner, and that the PLR is more often affected in peripheral than central autonomic disorders (Bremner and Smith, 2006). Whether the PLR dysfunction in PD is related to pre- or postganglionic dysfunction is unclear, although ANS symptoms in general are attributed to postganglionic dysfunction in PD (Druschky et al., 2000). It is, however, possible that visual disturbances in PD patients are partly attributed to PLR dysfunction which impairs optimal visual performance.

Disease duration did not differ in patients with PD and OH vs. PD without OH, however, patients with OH had higher LEDD and UPDRS scores, indicating more severe symptom severity, despite similar disease duration. In a previous study, in which patients with longer disease duration and symptom severity were compared to early-stage patients with PD, pupil constriction velocity was strongly and negatively correlated to advanced disease stage (You et al., 2021). Comparison to our data is difficult, as the patient group in our study had early-stage PD, and neither LEDD score nor UPDRS scores were associated with PLR measurements, which argues against confounding of disease severity.

The interdependence between constriction amplitude and peak constriction velocity has previously been highlighted when studying the PLR in healthy individuals, where the investigators used a light stimulus with a duration of 1 s, similar to our “long” paradigm (Bremner, 2012). In line with this study, we observed a strong correlation between constriction amplitude and peak constriction velocity in healthy individuals as well as in PD, both in the whole group as well as in the subgroups.

The pupil reaction in darkness causes a biphasic reaction: a rapid phase during which the parasympathetic drive is released from the iris sphincter, and a second phase where the sympathetic is responsible for the dilation (Bremner, 2009). After a short light stimulus, normal pupils are expected to recover to 90% within 5–6 s, while it would take 12–15 s to reach the original diameter (Pilley and Thompson, 1975). Regarding the role of the sympathetic ANS, we observed that T3/4 redilatation time (time to recover/redilate to 75% of the pupil diameter at stimulus onset) that has previously been used to assess the sympathetic function of the iris (Bremner and Smith, 2006), did not differ between HC and PD. However, dilation velocity and time to peak dilation velocity were compromised with increasing age. In line with this observation, a previous study of young and healthy volunteers showed that increasing age was inversely associated with pupil resting diameter, and with constriction and dilation velocities (Winn et al., 1994; Tekin et al., 2018).

The normal pupil requires approximately 250 ms to initiate constriction and reaches maximal miosis in approximately 1 s (Bremner, 2009). Our results indicate a prolonged latency in miosis in the long-flash paradigm compared to the short-flash condition for both groups, HC and PD. While in the short-flash paradigm the constriction initiates 250 ms after the light pulse offset, the constriction initiates while the light pulse is still on during the long-flash paradigm with a considerably increased latency of 371 ms in HC and 560 ms in the PD group, respectively (Table 2). It is difficult to explain this difference, but an impression is made that constriction initiates after stimulus offset in the short flash paradigm and awaits for the long light pulse offset, to a certain extent.

Our study has several limitations. Detailed examination of the autonomic functions was not performed, and the presence of OH was used as indicator of dysautonomia, which may have led to classification bias. The reason for that was that the PLR project was part of a bigger study that aimed to compare eye-movement parameters between PD and HC. However, such misclassification would result in underestimation of true differences between groups, rather than false positive correlations. Moreover, we acknowledge the fact that dilatation and constriction might be influenced by the initial pupil size. Our groups did not differ regarding the pupil size right before the stimulus period. Measurements at 15 s after each stimulus, i.e., exactly before the next flash, would provide more information on the pre-stimulus pupil size. In addition to that, and given that pupils can be spared in generalized disorders of the ANS, it is difficult to draw conclusions on the factors that affect the ANS and predict its involvement. Our results are interesting in that they indicate that parasympathetic functions can be associated to the disease, in PD, and become further pronounced in the presence of OH, whereas age and sex are stronger predictors of sympathetic functions and blink frequency, respectively. Also, all participants with PD were treated with dopaminergic agents and were tested in ON medication state. Despite the fact that we did not observe any correlation of LEDD score with PLR parameters, measurements in both ON and OFF medication state would be valuable to assess the influence of treatment on our results and their interpretation. It has, indeed, previously been shown that levodopa dosage correlates with pupillometric parameters in PD and may be used as a tool for non-invasive evaluation of the peripheral effect of levodopa (Bartošová et al., 2018).

## 5. Conclusion

Visual abnormalities in PD can be attributed to various factors, including defective PLR. PD patients that suffer peripheral autonomic dysfunction may have greater PLR defects, but the mechanisms by which the two entities are related cannot be explained by the present study. Using the correlation between peak constriction velocity and constriction amplitude could additionally be used to evaluate PLR together with other parameters of pupil dynamics.

## Data availability statement

The raw data supporting the conclusions of this article will be made available by the authors, without undue reservation.

## Ethics statement

The studies involving humans were approved by Stockholm Ethical Committee (DNR: 2018/437-31/2). The studies were conducted in accordance with the local legislation and institutional requirements. The participants provided their written informed consent to participate in this study.

## Author contributions

PT involved in the conception, organization, and execution of the research project, design, execution, and review and critique of the statistical analysis, and writing of the first draft and, review and critique of the manuscript preparation. MN involved in the conception, organization, and execution of the research project, review and critique of the statistical analysis and manuscript preparation. JW involved in the execution, and review and critique of the statistical analysis and manuscript preparation. GÖ and OL involved in the conception and organization of the research project, and review and critique of the manuscript preparation. PS involved in the conception and organization of the research project, and the review and critique of the manuscript preparation. IM involved in the conception, organization, execution of the research project, design, execution, review and critique of the statistical analysis, writing of the first draft, and review and critique of the manuscript preparation. All authors contributed to the article and approved the submitted version.
