# Supplementary material for: Pupil light reflex dynamics in Parkinson’s disease
Source: Front Integr Neurosci. 2023 Aug 31;17:1249554. doi: 10.3389/fnint.2023.1249554 (PMC10506153; doi:10.3389/fnint.2023.1249554)
Supplement: Supplementary file 1 [file Data_Sheet_1.PDF]

Healthy Control

Condition: Short flash

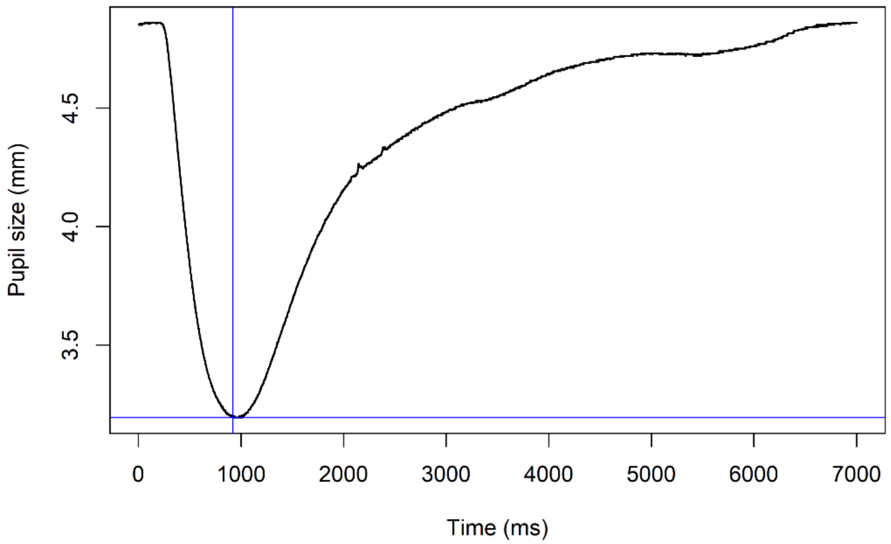

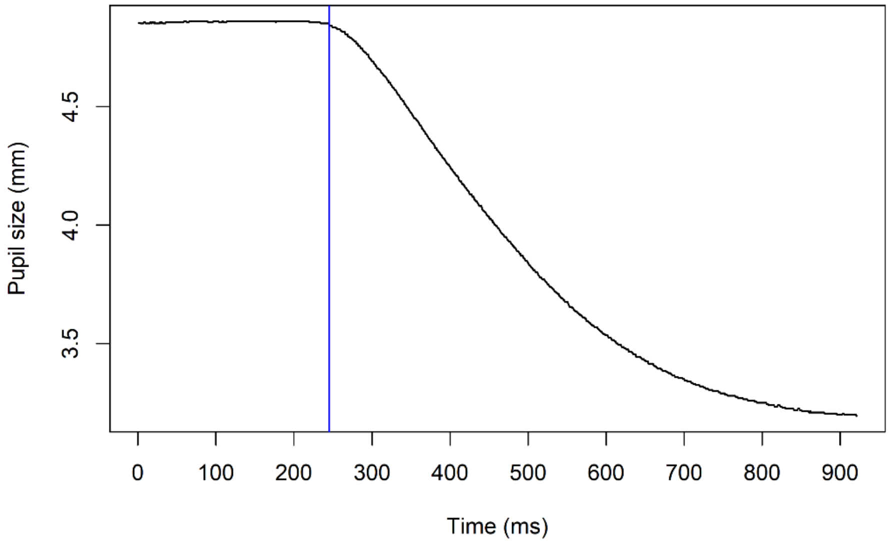

Condition: Long flash

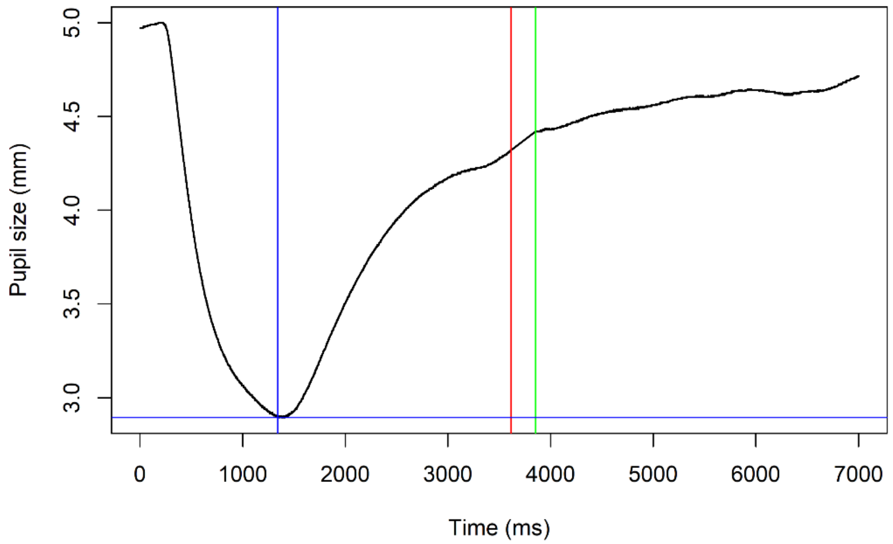

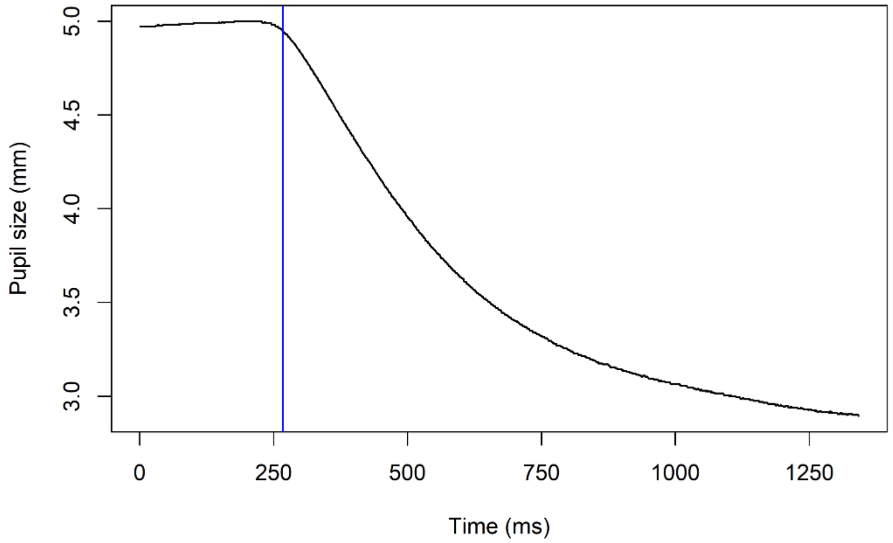

Parkinson's Disease patient

Condition: Short flash

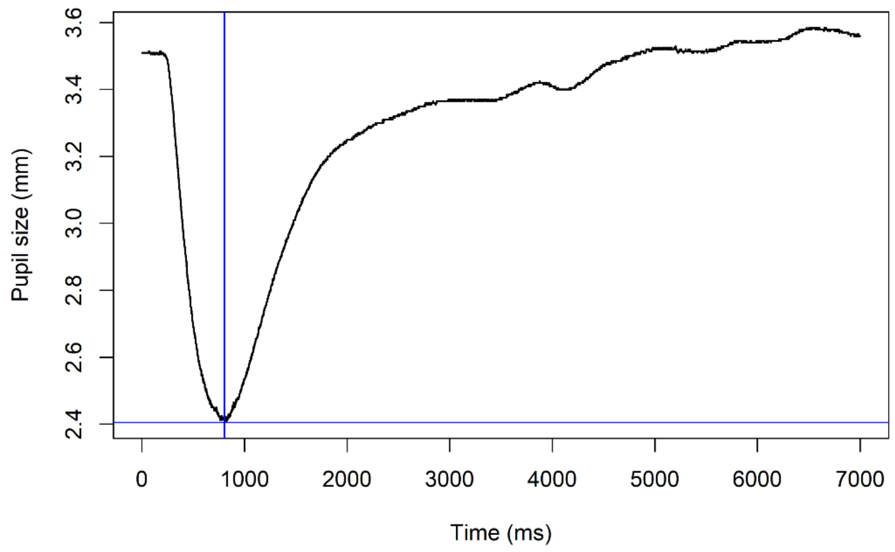

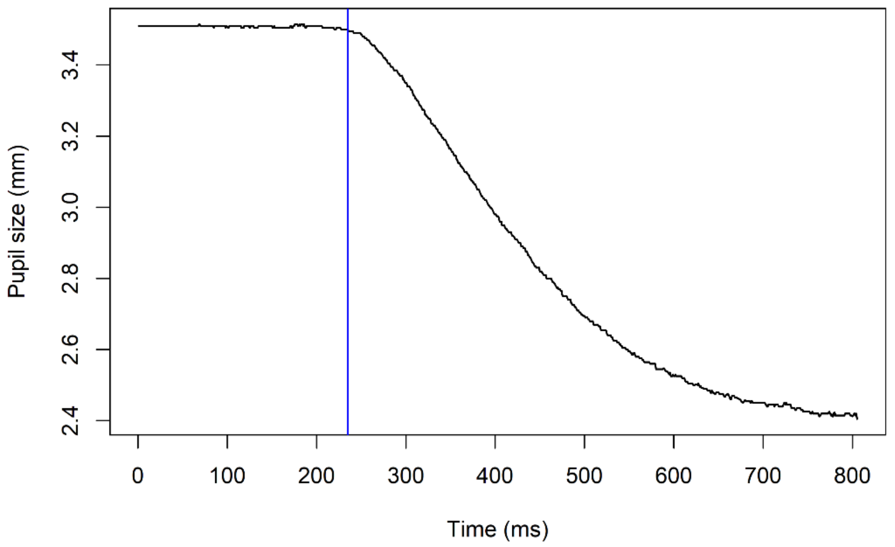

Condition: Long flash

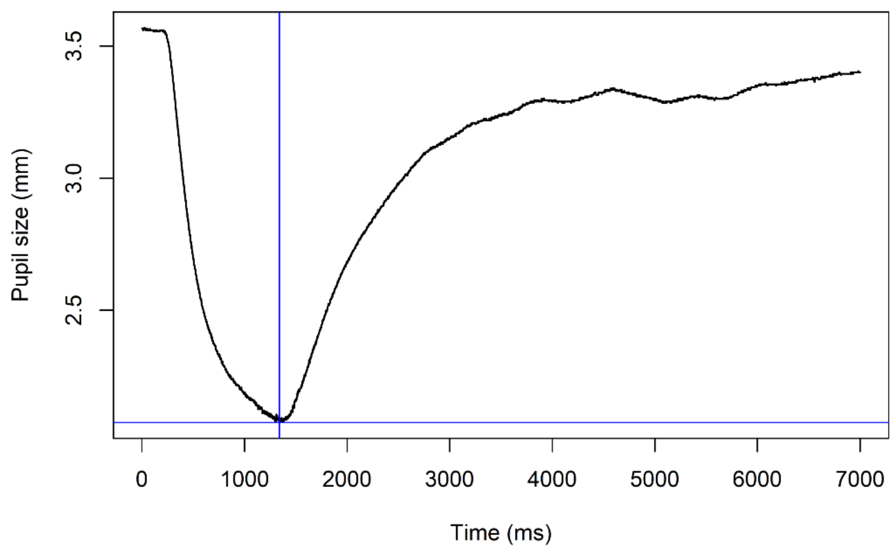

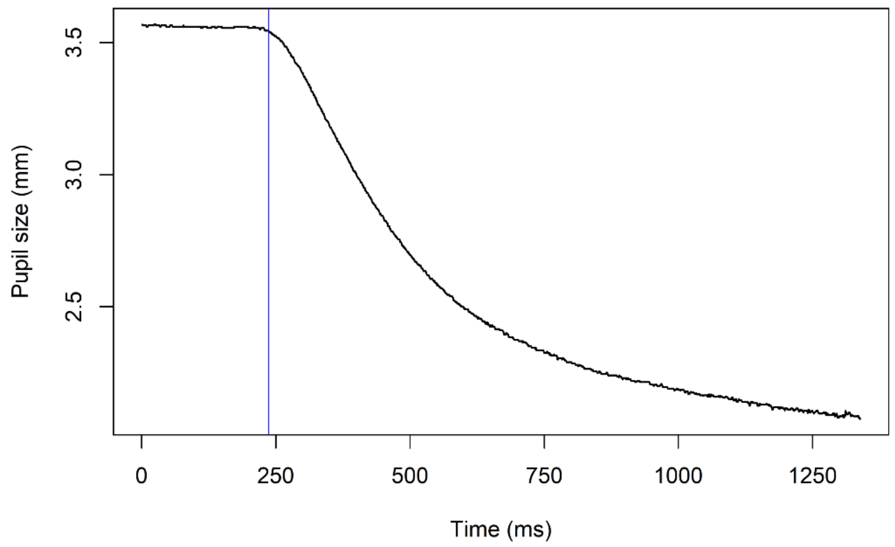

Picture 1. Plots in R base graphics showing a short (100 ms "flash") and long (1000 ms "flash") response for one PD and one HC subject. The examples are taken from good quality data without any or only a few missing samples (missing samples are interpolated and indicated by the interval between red and green vertical lines).There is also an additional plot zooming in on the early period from stimulus onset to maximum pupil constriction, essentially to get a clearer view of the latency period estimated by the change-point detection model we applied to the data. The plots are based on the processed eye tracking data (after applying a 5-sample running median).
