# Supplementary material for: Pupil light reflex dynamics in Parkinson’s disease
Source: Front Integr Neurosci. 2023 Aug 31;17:1249554. doi: 10.3389/fnint.2023.1249554 (PMC10506153; doi:10.3389/fnint.2023.1249554)
Supplement: Supplementary file 2 [file Table_1.DOCX]

List of computed parameters

1. Prestimulus pupildiameter mean: Mean pupil size in the prestimulus period (mm).
2. Prestimulus pupil diameter sd: Standard deviation of the pupil size in the prestimulus period (mm).
3. Pupil diameter mean: Mean pupil size in the stimulus-response period (mm).
4. Pupil diameter sd: Standard deviation of the pupil size in the stimulus-response period (mm).
5. Latency: Statistical approximation of constriction latency, i.e. the time between the stimulus onset and initiation of the response (ms). The constriction latency is estimated using a change-point detection model (Killick & Eckley, 2014) which identifies the first point in time at which the statistical properties (mean and variance) of the signal changes significantly.
6. Minimum pupil diameter: The pupil diameter at maximum constriction following stimulus onset (mm).
7. Time to max constriction: The time from stimulus onset to maximum pupil constriction (ms).
8. Constriction amplitude: The difference in pupil diameter between stimulus onset and maximum constriction (mm).
9. Constriction amplitude percentage change: The constriction amplitude given in percentage change (%).
10. Constriction velocity: The average velocity of the constricting movement (mm/s).
11. Peak constriction velocity: The velocity of the constricting movement when it is moving at its fastest speed (mm/s).
12. Time to peak constriction velocity: The time from stimulus onset until pupil constriction reaches peak velocity (ms).
13. Constriction slope: Linear slope coefficient, i.e., the estimated rate of change in pupil size per sample unit increase over time (1 sample = 0.83 ms) from stimulus onset to maximum constriction (mm).
14. Constriction intercept: The intercept for the linear slope coefficient (C27). Estimated pupil size (𝑦) at time 𝑡 is given by the formula 𝑦=𝑎+𝑏(𝑡∗(𝑓1000)), where 𝑎 is the constriction intercept, 𝑏 is the constriction slope, 𝑡 is the time point in milliseconds, and 𝑓 is the sampling frequency of the eye tracker (1200 Hz).
15. Dilation amplitude: The difference in pupil diameter between the beginning of dilation (i.e. where pupil diameter is at max constriction) and the end of the analysis window (mm).
16. Dilation amplitude percentage change: The dilation amplitude given in percentage change (%).
17. Dilation velocity: The average velocity of the dilating movement (mm/s).
18. Peak dilation velocity: The velocity of the dilating movement when it is moving at its fastest speed (mm/s).
19. Time to peak dilation velocity: The time from maximum constriction until pupil dilation reaches peak velocity (ms).
20. Dilation slope: Linear slope coefficient, i.e., the estimated rate of change in pupil size per sample unit increase over time (1 sample = 0.83 ms) from the beginning of dilation (maximum constriction) to the end of the analysis window (mm).
21. Dilation intercept: The intercept for the linear slope coefficient (C34). Estimated pupil size (𝑦) at time 𝑡 is given by the formula 𝑦=𝑎+𝑏(𝑡∗(𝑓1000)), where 𝑎 is the dilation intercept, 𝑏 is the dilation slope, 𝑡 is the time point in milliseconds, and 𝑓 is the sampling frequency of the eye tracker (1200 Hz).
22. Dilation recovery time 25p: The time to recover/redilate to 25% of the pupil diameter at stimulus onset (ms).
23. Dilation recovery time 50p: The time to recover/redilate to 50% of the pupil diameter at stimulus onset (ms).
24. Dilation recovery time 75p: The time to recover/redilate to 75% of the pupil diameter at stimulus onset (ms).
25. Poststimulus pupil diameter 6s: The pupil diameter at six seconds following stimulus offset (mm).
26. Blink frequency: Approximate frequency of blinks during the stimulus-response period (blinks/minute).

Killick, R., Eckley, I.A. (2014) changepoint: An R package for changepoint analysis. *Journal of Statistical Software* 58(3) 1-19.

Supplementary table 1: Pupil response metrics recorded after the short (100msec) flash stimulus. Comparisons between HC and PD.

|  | HC | PD | P |
| --- | --- | --- | --- |
|  | Median/IQR | Median/IQR |  |
| Short flash |  |  |  |
| Prestimulus pupil diameter mean | 4.4/1.01 | 3.87/1.3 | 0.06 |
| Prestimulus pupil diameter SD | 0.0114/0.02 | 0.116/0.02 | 0.9 |
| Pupil diameter mean | 3.9/0.76 | 3.6/0.89 | 0.1 |
| Pupil diameter DS | 0.41/0.13 | 0.34/0.14 | 0.08 |
| Latency | 249.17/38.5 | 252.9/71.25 | 0.25 |
| Minimum pupil diameter | 2.82/0.53 | 2.6/0.62 | 0.3 |
| Time to max constriction | 814.38/91.25 | 785/99.38 | 0.25 |
| Constriction amplitude | -1.49/0.37 | -1.2/0.58 | **0.046** |
| Constriction amplitude percentage change | -34.27/5.58 | -32/7.63 | 0.09 |
| Constriction velocity | -1.8/0.44 | -1.53/0.45 | 0.06 |
| Peak constriction velocity | -4.85/1.2 | -4.24/1 | **0.02** |
| Time to peak constriction velocity | 382.29/47.92 | 393.54/42.5 | 0.17 |
| Constriction slope | -0.002/0 | -0.0017/0 | **0.047** |
| Constriction intercept | 4.49/0.82 | 4/1.3 | 0.2 |
| Dilation amplitude | 1.46/0.41 | 1.14/0.47 | **0.03** |
| Dilation amplitude percentage change | 49.345/10 | 45.03/18.3 | 0.09 |
| Dilation velocity | 0.24/0.07 | 0.2/0.08 | **0.027** |
| Peak dilation velocity | 1.5/0.44 | 1.41/0.57 | 0.6 |
| Time to peak dilation velocity | 403.75/93.5 | 432.9/1593 | 0.5 |
| Dilation slope | 0.0001/0 | 0.0001/0 | 0.13 |
| Dilation intercept | 3.45/0.6 | 3.2/0.66 | 0.1 |
| Dilation recovery time 25p | 413.8/92.71 | 383.75/95.8 | 0.4 |
| Dilation recovery time 5p | 739.17/234.6 | 680.4/191.25 | 0.4 |
| Dilation recovery time 75p | 1603.75/1120 | 1351/711.4 | 0.14 |
| Poststimulus pupil diameter 6s | 4.11/0.88 | 3.84/1.06 | 0.5 |
| Prestimulus blink frequency | 17.14/10.7 | 8.57/32.14 | 0.8 |
| Blink frequency | 16.87/8.47 | 8.45/28.5 | 0.9 |

HC: Healthy Controls, PD: Parkinson’s Disease, IQR: Interquartile Range, sd: standard deviation. Statistical significant (p<0.05) results are highlighted in black. Pupil diameter, constriction/dilation amplitude, and poststimulus pupil diameter 6s, are computed in mm. Latency, time to max constriction, and time to max constriction/dilation velocity are computed in msec. Velocities are computed in mm/sec. Dilation recovery time 25/50/75 p is computed in msec. Blink frequency is computed in blinks/ min.

Supplementary Table 2. Pupil response metrics recorded after the long (1000msec) flash stimulus. Comparisons between HC and PD.

|  | HC | PD | P |
| --- | --- | --- | --- |
|  | Median/IQR | Median/IQR |  |
| Long flash |  |  |  |
| Prestimulus pupil diameter mean | 4.4/1.01 | 3.87/1.3 | 0.06 |
| Prestimulus pupil diameter SD | 0.0114/0.02 | 0.0116/0.02 | 0.9 |
| Pupil diameter mean | 3.66/0.65 | 3.25/0.89 | 0.1 |
| Pupil diameter SD | 0.52/0.18 | 0.42/0.18 | **0.048** |
| Latency | 371/379 | 573.75/319 | 0.08 |
| Minimum pupil diameter | 2.4/0.39 | 2.34/0.42 | 0.3 |
| Time to max constriction | 1343/105.6 | 1313/140 | 0.7 |
| Constriction amplitude | -1.8/0.65 | -1.5/0.83 | 0.07 |
| Constriction amplitude percentage change | -42.92/9.44 | -38.8/9.23 | 0.1 |
| Constriction velocity | -1.47/0.44 | -1.2/0.62 | **0.028** |
| Peak constriction velocity | -4.8/1.09 | -4.05/1.24 | **0.002** |
| Time to peak constriction velocity | 387.9/46.04 | 405/73.12 | 0.07 |
| Constriction slope | -0.0015/0 | -0.0013/0 | **0.012** |
| Constriction intercept | 4.34/0.98 | 3.72/1.35 | 0.074 |
| Dilation amplitude | 1.68/0.59 | 1.39/0.59 | **0.024** |
| Dilation amplitude percentage change | 66.9/18.17 | 55.5/22.96 | 0.06 |
| Dilation velocity | 0.26/0.09 | 0.21/0.09 | **0.036** |
| Peak dilation velocity | 1.54/0.58 | 1.53/0.57 | 0.98 |
| Time to peak dilation velocity | 463.75/142.71 | 482.92/1861 | 0.4 |
| Dilation slope | 0.0002/0 | 0.0001/0 | 0.063 |
| Dilation intercept | 3.11/0.51 | 2.88/0.57 | 0.1 |
| Dilation recovery time 25p | 509.17/132.7 | 499.6/137.7 | 0.5 |
| Dilation recovery time 50p | 1013.5/477 | 990.8/436.2 | 0.4 |
| Dilation recovery time 75p | 2466/1740 | 2320.82/1632.5 | 0.7 |
| Poststimulus pupil diameter 6 s | 4.08/0.87 | 3.7/1.07 | 0.13 |
| Prestimulus blink frequency | 17.14/10.7 | 8.57/32.14 | 0.8 |
| Blink frequency | 14.99/15 | 14.99/22.55 | 0.8 |

HC: Healthy Controls, PD: Parkinson’s Disease, IQR: Interquartile Range, sd: standard deviation. Statistical significant (p<0.05) results are highlighted in black. Pupil diameter, constriction/dilation amplitude, and poststimulus pupil diameter 6s, are computed in mm. Latency, time to max constriction, and time to max constriction/dilation velocity are computed in msec. Velocities are computed in mm/sec. Dilation recovery time 25/50/75 p is computed in msec. Blink frequency is computed in blinks/ min.
